# Effects of light spectra on morphological characteristics, primary and specialized metabolites of *Thymus vulgaris* L^☆^

**DOI:** 10.1016/j.heliyon.2023.e23032

**Published:** 2023-12-01

**Authors:** Forouh Sadat Seyedi, Mehdi Ghasemi Nafchi, Saeed Reezi

**Affiliations:** Department of Horticulture Science, College of Agriculture, Shahrekord University, Iran

**Keywords:** Antioxidant activity, LED, Light, Primary metabolites, *Thymus vulgaris*, Volatile compound

## Abstract

Light is a crucial environmental factor that profoundly influences the growth and development of plants. However, the precise mechanisms by which light affects biochemical processes and growth and development factors in *Thymus vulgaris* remain unknown, necessitating further investigation. Hence, this study aimed to investigate the impact of different light spectra, including red, blue, red-blue, and white lights, on the morphological characteristics, primary, and specialized metabolites of *T. vulgaris*. Compared to white light, red light significantly increased leaf area (by 64 %), the number of branches (by 132 %), and dry weight (by 6.2 %), although a 40 % reduction in fresh weight was observed under red light conditions. Red-blue light notably enhanced canopy width, fresh weight, and dry weight. Gas chromatography/mass spectrometry (GC/MS) analysis of the plant's essential oil (EO) revealed that *p*-Cymene and *γ*-Terpinene were present at the highest levels. Notably, *p*-Cymene exhibited the highest concentrations under white light and blue light treatments, reaching 60.92 % and 59.53 %, respectively. Moreover, under the same light conditions, phenol and antioxidant levels were significantly elevated. Overall, these findings indicate that red and red-blue light spectra are the most favorable for thyme production.

## Graphical abstract

**Figure:**
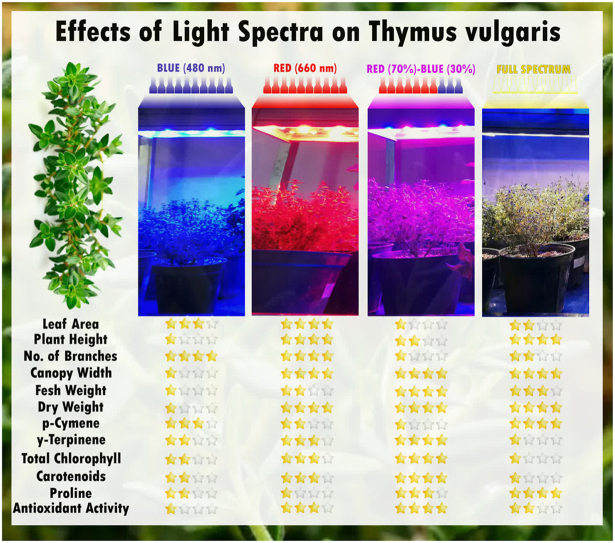

## 1 Introduction

Environmental conditions, including light, humidity, and temperature, have a direct impact on plant growth and development. Among these factors, light plays a crucial role in shaping desirable morphological and photosynthetic characteristics. Light intensity and quality control plant responses, including phytochemical production and physiological processes, in a spectrum-dependent manner [1]. Artificial light can be used as a single or complementary light source in deficient light times. Light sources such as fluorescent lamps, metal halide, sodium superphosphates, and neon lamps are commonly used in plant cultivation [2]. Artificial light sources such as fluorescent lamps, metal halide, sodium superphosphates, and neon lamps are commonly used in plant cultivation to supplement deficient light conditions. However, these lamps often emit unnecessary wavelengths outside the photosynthetically active radiation range and can adversely affect growth quality [3].

In this context, light-emitting diodes (LEDs) have emerged as low-cost and compact light sources that emit minimal heat. They offer adjustable light intensity and appropriate wavelengths, making them suitable for various plant species. LEDs also provide advantages in reducing oxidative stress caused by excessive light energy. The wide range of available LED colors allows for precise wavelength control without the need for additional filters [4]. Applying the combination of red and blue LED lamps has proved to enhance the yield of many plant species rather than using broad-band light deficiently filtered in blue or red [5]. Studies have demonstrated that red, blue, and ultraviolet (UV) light can increase fresh weight, photosynthetic activity, energy consumption efficiency, essential oil (EO) content, phenolic compounds, and antioxidant capacity in various plants compared to white light or sunlight, although the specific effects vary among species and light treatments [2,6].

*Thymus vulgaris* L. is one of the valuable genera of the mint family (Lamiaceae), originally found in the western Mediterranean region and widely distributed in Asia, Europe, and Africa [7]. Due to its aromatic properties, unique essential oil compounds, and diverse pharmaceutical and industrial applications, *T. vulgaris* is commonly used as a herbal tea, flavoring agent, and medicinal plant [8]. Nowadays, there is an enormous requirement for thyme-derived products around the globe [9]. By investigating the impact of light on *T. vulgaris*, researchers can gain valuable knowledge to optimize cultivation techniques, enhance crop productivity, and improve the quality of thyme-derived products.

Blue light is a critical environmental factor that profoundly influences various aspects of plant growth and development. It regulates photomorphogenesis, including seedling growth, leaf expansion, and chloroplast development, thereby shaping plant architecture [10,11]. Additionally, blue light plays a role in stomatal regulation, affecting gas exchange and water loss [12]. It is essential for chlorophyll synthesis, regulation of flowering time, secondary metabolite biosynthesis, entrainment of the circadian rhythm, and activation of photoreceptor proteins [13,14]. Cryptochromes and phototropins are key photoreceptor proteins that mediate the effects of blue light within plant cells [15].

Red light exerts diverse effects on plants and plays a pivotal role in physiological processes. It is essential for photosynthesis, facilitating energy generation and carbohydrate production required for plant growth [16]. Moreover, red light stimulates leaf expansion and stem elongation, enabling plants to optimize light absorption and efficiently navigate crowded environments [17]. These effects are mediated by phytochromes, photoreceptor proteins that govern processes such as seed germination, stem elongation, and gene expression [18].

The combination of blue and red light has significant effects on plant growth. The ratio of blue to red light profoundly influences plant morphology, pigmentation, and overall growth patterns. This light combination offers high energy efficiency in terms of plant absorption, making it an attractive option for artificial lighting systems. However, it is important to note that the optimal blue-to-red light ratio varies significantly among plant species and growth stages. Each plant has specific light requirements, and different growth stages, such as vegetative or flowering, may necessitate varying ratios. Therefore, understanding the precise light preferences of the target plant is crucial for achieving optimal results in terms of morphology, pigmentation, chemical compounds, and overall growth patterns [19].

LED light sources have been successfully used to grow various types of herbal and medicinal plants [20,21]. This study aims to analyze the effects of different light spectra on the quantitative and qualitative characteristics of *T. vulgaris*. Blue, red, blue-red and, white spectra were used to investigate their impact on essential oil compounds, antioxidant activity, anthocyanin content, phenols, and morphological characteristics of *T. vulgaris*.

## 2 Materials and methods

### 2.1 Plant material and growth condition

This experiment was completely performed in a randomized design with three replications. Each growth chamber contained six pots of *T. Vulgaris* classified as chemotype I, known for its high thymol content [22]. The seedlings were purchased from Fereydan Green Plant Company (approved by the Iranian government), and then the plants were transplanted to the pots (15 × 15 cm in size) containing cocopeat and perlite in a 2:1 ratio. The seedlings were pruned to a height of 5 cm and acclimated to the new growing conditions in a research greenhouse maintained at a temperature of +24 °C. Daily irrigation was accomplished for two weeks to allow the plants adapt to the new growing conditions. After the acclimation period, the plants were transferred to four growth chambers equipped with LED lamps and controlled environmental conditions (40–50 % humidity and 23 ± 2 °C temperature). The irrigation was carried out every three days, once a week with a nutrient solution that was determined by HydroBuddy software, version 1.4. The nutrient solution had a pH value of 5.9 ± 0.3 and an electrical conductivity (EC) of 1.2 mScm^−1^. Essential macro and micronutrients necessary for optimal growth and development of *T. vulgaris* were included in the nutrient solution. These nutrients, such as nitrogen (N), phosphorus (P), potassium (K), calcium (Ca), magnesium (Mg), iron (Fe), manganese (Mn), zinc (Zn), copper (Cu), molybdenum (Mo), and boron (B), were sourced from reputable companies like Tradecorp (Spain) and Yara (Norway). The concentrations of these nutrients were adjusted based on the software's recommendations and the specific requirements of *T. vulgaris*. To ensure equal light exposure for all plants, the pots were rotated and moved horizontally every four days. Air circulation within the growth chambers was maintained using fans installed in each chamber. After a period of 60 days, the plants were harvested by removing the upper two-thirds of the plants and then dried at 25 °C in a shaded area.

### 2.2 Methods

#### 2.2.1 Illumination treatments

**Fig. 1:**
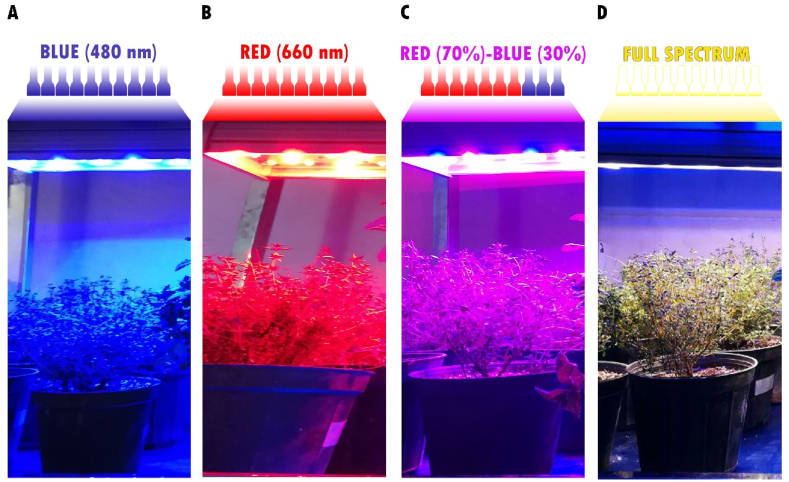
Experimental arrangement featuring growth chambers containing diverse LED arrays and *T*.*vulgaris* pots subjected to various light treatments. These growth chambers are outfitted with distinct LED arrays as blue (A), red (B), red + blue (C), and full spectrum (D) LED.

The growth chambers were equipped with monochromatic LED lights in blue (480 nm) (Fig. 1A), red (660 nm) (Fig. 1B), a combination of red (70 %) and blue (30 %) (Fig. 1C) and full spectrum light (Fig. 1D), which were purchased from Golnoor Company (Isfahan, Iran). The white light spectrum (Peak at 570 nm) was also used as the control. To prevent the entry of sunlight, the growth chambers were covered with dark black polyethylene sheets. The photosynthetic photon flux density (PPFD) was set at 120 ± 20 μmol (photon) m^−2^s^−1^ (equivalent to 552 ± 92 lux), and the light/dark cycle was maintained at 16 h of light and 8 h of darkness throughout the 60-day experimental period. Photosynthetic active radiation (PAR), using a quantum sensor (Model MQ-100, apogee instruments, USA) and light spectra, using a portable spectroradiometer (EPP 2000C, Stellar Net, Inc., USA) placed at a distance of 40 cm from the LED lamps and at the plant's canopy surface, were measured. Along with the experiments, the fixtures were regulated to keep the plant's canopy at a distance of 40 cm from the light source.

### 2.3 Measurements and analyses

#### 2.3.1 Morphological traits

The adult leaves located in the middle part of the plant were randomly harvested. The length and width of the leaves were measured using a digital caliper (Digital caliper Guanglu, 0–100 mm). The leaf area was calculated using Digimizer software. In order to measure the internode length, the distance between the second and the third nodes was considered, and in terms of the stem diameter measurement, the last one-third of the main stem [23] was calculated by the digital caliper. The number of all branches was counted, and the canopy height was measured using a ruler with an accuracy of 1 mm. The fresh weight of whole canopies was recorded with a digital scale (Citizen CY 220) with an accuracy of 0.001 g. Next, they were dried under the shade at the temperature of 25 °C, and the dry weight was measured.

#### 2.3.2 Phytochemical traits

##### 2.3.2.1 Preparation of plant extract

To prepare the dry hydroalcoholic extract, the samples were soaked in 70 % alcohol for 72 h. After this period, the extract was passed through the filter paper and dried in an incubator at 38 °C [24].

##### 2.3.2.2 Volatile compounds measurement

The Headspace technique was applied to extract volatile compounds. Also, gas chromatography (GC) and gas chromatography/mass spectrometry (GC/MS) were used to isolate and detect the compounds. In this method, 2 g of each dried sample was powdered, dispersed, and then added to vials (with a volume of 20 ml), and were quickly covered with a rubber layer made from silicon, and an aluminum cap. Then, the vials were transferred to the headspace tray. The headspace was moved into a CombiPAL system equipped with an automatic sampler, heater, and stringer. The vials were heated up to 80 °C and kept at this temperature for 20 min while stirring. The sampling needle and transmission line were maintained at a temperature above 85 °C.

The GC/MS analysis was performed using an Agilent 5975C model with an ion trap system. The HP-5MS capillary column, with a length of 30 cm, an internal diameter of 0.25 mm, and a static phase layer thickness of 0.25 μm, was used. The thermal program was set from 40 to 240 °C at a rate of 4 °C/min. The injection chamber and linear transfer temperature were regulated at 260 °C. Volatile compounds were identified based on the inhibition time of the compounds, the mass spectrometer inhibition index, and finally the comparison of these parameters with the standard compounds and available information on NIST, and Adams spectrometer data banks.

##### 2.3.2.3 Total phenol content

The total phenolic content (TPC) was determined using the Folin–Ciocalteu colorimetric method with gallic acid as the standard. Initially, 0.01 g of the dried extract was dissolved in 10 ml of 60 % methanol. Then, 0.1 ml of the extract was transferred to a test tube, and 0.5 ml of 10 % Folin–Ciocalteu reagent was added. After three to 8 min, 0.4 ml of 7.5 % sodium carbonate solution was added to the tube. The mixture was kept at the laboratory temperature for 30 min, and the optical absorption was measured at a wavelength of 765 nm using a spectrophotometer (Jenway 6320D) against a blank. All measurements were carried out in triplicate. Simultaneously, different dilutions of gallic acid were prepared, and a standard curve was constructed (Y = 0.0182x-0.0142). The total phenol content of the extract was then reported as milligram of gallic acid per gram of the dried extract [25].

##### 2.3.2.4 Total flavonoid content

The total flavonoid content was determined using a slightly modified aluminum chloride colorimetry method as previously described [25]. An aliquot of 0.01 g of the dried extract was dissolved in 10 ml of 60 % methanol. Next, 0.1 ml of this solution was transferred to a test tube and mixed with a solution containing 0.5 ml of 2 % aluminum chloride (AlCl3) and 3 ml of 5 % potassium acetate (CH3CO2K). After 40 min, the absorption of the samples was measured at 415 nm. All determinations were accomplished in triplicate. Simultaneously, different dilutions of quercetin were prepared and the test was performed and the curve was designed exactly following the above-mentioned procedure. The absorption value of the samples was put up in the standard chart, and the total flavonoid content was calculated based on the linear equation of quercetin standard curve (Y = 0.0025x+0.0235) and expressed as the milligram of quercetin per gram of the dried extract.

##### 2.3.2.5 Antioxidant activity

The antioxidant activity was measured using the 2,2-diphenyl-1-picrylhydrazyl (DPPH) based on free radical inhibition. Firstly, different concentrations of the extract were prepared. Then, 200 μL of each concentration was mixed with one ml of 90 μM DPPH solution. After that, they reached a volume of four ml with 95 % methanol and were shaken in the dark using a shaker at 25 °C for 60 min. The absorbance of the samples and the control (methanol and DPPH) were read at a wavelength of 517 nm using a spectrophotometer. All determinations were carried out in triplicate. Eventually, the free radical inhibition values were calculated using formula I = 100*((control absorption -Absorbance of the sample)/control absorption) [20].

##### 2.3.2.6 Anthocyanin

To determine the amount of anthocyanin, 0.06–0.08 g of the dried extract was dissolved in 10 ml of distilled water to prepare the extracted stock. Then, two buffers, one with pH = 1 and the other with pH = 4.5 were provided. To arrange the buffer with pH = 1, 1.86 g of potassium chloride salt was dissolved in distilled water, and the pH was adjusted at 1 by adding HCL acid. Subsequently, the volume of the buffer was increased to 1000 ml. To organize the buffer with pH = 4.5, 54.43 g of sodium acetate salt was dissolved in distilled water, and the pH was adjusted at 4.5 by adding HCL acid. The volume of the buffer was increased to 1000 ml. In the next step, 5 ml of the stock prepared from the extract was taken and diluted to a volume of 25 ml using the pH = 1 buffer (the same procedure was followed for the pH = 4.5 buffer). All extractions were kept in the dark for 10 min. The optical absorption was measured at 520 and 700 nm [26]. All determinations were executed in a triplicate manner. Finally, the calculations were performed using the following formulas.

A=(A520‐A700)‐pH=1(A520‐A700)pH=4.5

C=(A×MW×DF×V×1000)/(E×L×M)

A520 and A700: Absorbance values at λ = 520 and 700 nm. MW: Molecular weight of the dominant anthocyanin (2.449). DF: Buffer-to-stock ratio (5). E: Molar absorption (26900). L: 1. M: Extract amount (g). V: The volume of distilled water in which the extract was dissolved. C: the amount of anthocyanin (mg.g^−1^ Extract).

#### 2.3.3 Physiological traits

##### 2.3.3.1 Chlorophylls and carotenoids

The measurement of chlorophyll *a* (Chla), chlorophyll *b* (Chlb), total chlorophyll (TChl), and carotenoids was carried out using the Lichtenthaler method [27]. A randomly harvested mature and fresh leaf weighing 0.25 g was homogenized in a cold porcelain mortar with 10 ml of 80 % acetone until a homogeneous solution was obtained. Subsequently, the samples were transferred to centrifugal tubes and centrifuged at 3500 rpm for 10 min to separate the liquid from the solid components. The liquid portion was used for extraction, and the light absorption of the soluble fraction was measured at 663 nm, 646 nm, and 470 nm using a spectrophotometer. All determinations were executed in a triplicate manner. Consequently, concentrations of Chla, Chlb, TChl and carotenoids (mg/g FW) were calculated using the following formulas.

$$\text{Chla}\;(\text{mg}.\;g^{‐1}\text{FW})=\left(12.25\left(A663\right)‐2.79\left(A646\right)\right)\times V/100\times W$$

$$\text{Chlb}\;(\text{mg}.\;g^{‐1}\text{FW})=\left(25.51\left(A646\right)‐5.10\left(A663\right)\right)\times V/100\times W$$

$$\text{TChl}\;(\text{mg}.\;g^{‐1}\;\text{FW})=\text{Chla}+\text{Chlb}$$

$$\text{Carotenoids}\;(\text{mg}.\;g^{‐1}\text{FW})=(\left(1000\left(A470\right)‐1.8\left(\text{Chla}\right)‐85.02\left(\text{Chlb}\right)/198\right)$$

A663, A645, and A470: Absorbance values at λ = 663, 645, and 470 nm. V: Amount of acetone consumed. W: fresh weight of the leaf sample (g).

##### 2.3.3.2 Soluble sugars

For the quantification of total soluble sugars (TSS), a 0.5 g sample of fresh leaves was crushed and mixed with 10 ml of 95 % ethanol. The samples were then centrifuged at 3500 rpm for 10 min. Thereafter, 0.1 ml of the alcoholic extract was combined with 3 ml of anthrone solution (prepared by mixing 200 mg of anthrone with 100 ml of 72 % H2SO4) and heated in a boiling water bath for 10 min. After cooling, the absorbance was measured at 625 nm [28].

##### 2.3.3.3 Proline content

To measure proline content, two ml of the alcoholic extract (prepared for soluble sugars) was mixed with two ml of acidic ninhydrin solution. The acidic ninhydrin solution was prepared by pouing 1.25 g of ninhydrin in 30 ml of acetic acid and adding 20 ml of 6 M phosphoric acid. The mixture was then heated in a boiling water bath at 100 °C for 1 h. After complete cooling, four ml of toluene was added, and the solution was mixed and shaken for 3 min. Finally, the upper phase was separated, and the absorbance was read at 515 nm.

### 2.4 Statistical analysis

All data were analyzed using SAS software, and charts were plotted using Excel software. The mean comparisons were accomplished based on the Least Significant Differences (LSD) method and correlation analysis was performed using the Pearson test. The correlation graph was generated using RStudio software version 4.3.0. Additionally, the principal component analysis (PCA) was carried out using Prism software version 9.5.1.

## 3 Result and discussion

### 3.1 Light quality effects on morphological traits

In accordance with the results, various light treatments showed significant differences in leaf area, number of branches, canopy width, and fresh and dry weight at a significance level of 0.01. However, there were no significant differences among the light treatments in terms of internode length, leaf length and width, number of nodes, stem length and diameter, as well as plant height (Table 1).

**Table 1:** The influence of different light colors on morphological traits of *Thymus vulgaris*.

Traits	unit	Light colors				
		Blue	Red	White	Red + Blue	CV
Leaf Length ^ns^	mm	7.65 ± 0.21	7.70 ± 0.49	6.84 ± 0.38	7.25 ± 0.47	6.70
Leaf Width ^ns^	mm	3.10 ± 0.38	3.28 ± 0.21	2.91 ± 0.29	3.01 ± 0.22	11.40
L/W ^ns^	–	2.50 ± 0.28	2.35 ± 0.01	2.36 ± 0.11	2.41 ± 0.5	7.80
Leaf Area **	mm^2^	0.19 ± 0.1^b^	0.28 ± 0.00^a^	0.17 ± 0.00^c^	0.12 ± 0.00^d^	3.10
Internode length ^ns^	mm	17.04 ± 1.43	20.44 ± 0.49	20.60 ± 1.18	18.32 ± 1.13	7.10
Number of Node ^ns^	–	7.00 ± 0.82	7.33 ± 0.94	7.67 ± 1.25	7.00 ± 0.82	16.40
Stem Length ^ns^	cm	9.22 ± 1.96	11.90 ± 2.06	10.98 ± 1.70	9.24 ± 1.21	20.90
Stem Diameter ^ns^	mm	0.85 ± 0.4	0.96 ± 0.14	0.89 ± 0.2	0.80 ± 0.04	10.42
Number of Branch **	–	66.00 ± 10.2^a^	58.33 ± 4.19^a^	25.33 ± 3.40^b^	24.00 ± 6.38^b^	18.60
Plant Height ^ns^	cm	17.50 ± 2.91	21.33 ± 2.72	21.67 ± 2.05	20.55 ± 2.86	16.10
Canopy width **	cm	17.39 ± 0.28^b^	22.25 ± 1.83^a^	23.52 ± 1.37^a^	23.71 ± 1.91^a^	8.40
Fresh Weight **	g	10.08 ± 0.37^d^	15.47 ± 1.13^c^	25.23 ± 1.38^b^	31.45 ± 1.69^a^	7.40
Dry Weight **	g	0.98 ± 0.06^b^	2.87 ± 0.21^a^	2.70 ± 0.28^a^	2.61 ± 0.30^a^	12.40

L/W: Leaf length/leaf width; Data are means ± SE (n = 3); ns: Values are not significantly different.

*: Values are significantly different at P ≥ 0.05.

**: Values are significantly different at P ≥ 0.01.

The values followed by the same alphabet do not significantly differ according to Fisher's Duncan test.

In our study, the highest leaf area of the samples was observed in the red light treatment, measuring 0.28 cm^2^, while the lowest leaf area was achieved under the red + blue light treatment measuring 0.12 cm^2^. Based on previous findings, the response of plant species to light quality is different; however, red and blue lights have generally the greatest effect on plant growth [29]. According to Lin et al. [2], the size of a leaf influences the speed of photosynthetic processes. Larger leaf area allows for increased light absorption, leading to a higher availability of energy for photosynthesis. This is because a larger surface area captures more light, enhancing the efficiency of the light-dependent reactions. Additionally, larger leaves facilitate better gas exchange, particularly the uptake of carbon dioxide, which is essential for photosynthesis [2]. Among the different light spectrums given to *Alternanthera brasiliana*, blue light caused the largest leaf area [30]. In *Chrysanthemum*, plants grown in red light had smaller leaves, whereas blue light promoted leaf development [31]. Similar results were observed in *Lactuca sativa* [32] and *Chrysanthemum* [33].

The white and red light treatments exhibited the longest internode lengths, measuring 20.59 mm and 20.44 mm, respectively, while the shortest internode length of 17.04 mm was observed under the blue light treatment. Similar morphological changes in *Stevia rebaodiana* have been reported previously [34], which indicated the attainment of the shortest stem under blue LED lamps. The highest number of branches was obtained in the blue (66 branches) and the red (55.3 branches) monochromatic light, with no significant difference between these two light qualities. Conversely, the lowest number of branches was recorded in the blue + red light treatment, with 24 branches. Blue light has been found to induce lateral branch formation and inhibit terminal bud growth [35]. No significant differences were detected among the different light spectrums in terms of plant height and statistically they were almost identical.

Cryptochromes, responsible for blue light absorption, play a notable role in preventing stem elongation [29]. Red light has been reported to have an increasing effect on stem length and number in grape genotypes [5]. *Tagetes erecta* and *Salvia militrohiza* plants exhibited greater height under blue light compared to other fluorescent treatments [36]. Also, Su et al. [37] indicated a negative effect of red light on the height of *cucumis sativus* seedlings. A comparison study on industrial hemp plants demonstrated that blue light significantly influenced the growth of *Cannabis sativa*, particularly in terms of fresh and dry stem weight, plant height, number of leaves per plant, and root length [38]. The length of the stem in tomato plants was affected by the amount of blue light in the studied treatments [39]. In addition, the reduction of *Cucumis sativus* height occurred under blue light conditions [40]. The use of red-blue light led to a decrease in *cannabis* plant development compared to the control, through the reduction of stem fresh weight, stem dry weight, number of leaves per plant, plant height, stem diameter, and root length [38]. Also in *Stevia rebaudiana*, blue light led to shorter stems [34].

The maximum canopy width was 23.71 cm, achieved under the white light treatment, and the lowest canopy width was 17.38 cm, obtained under the blue light treatment. The plant's response to the blue light condition included an increase in the number of branches and a reduction in canopy width, potentially indicating an adaptive mechanism to minimize light exposure and decrease evaporation rates from the leaf area, thus providing a form of protection [1,41,42].

Weight analysis revealed that the highest fresh weight of 31.45 g was obtained under the red + blue light treatment, whereas the lowest fresh weight of 10.08 g was recorded under the blue light treatment. Similarly, the red light treatment resulted in the highest dry weight of 2.87 g, while the lowest dry weight (0.97 g) was observed under the blue light treatment. In several studies conducted on *Triticum aestivum*, *Spinacea oleracea*, *Lactuca sativa*, and *Raphanus sativus*, plants grown under red light had lower dry weight compared to fluorescent lamps [[43], [44], [45]].

Red light has been found to have the most irritating effect on the weight and height of *Taraxacum officinale* [46] and *Rehmannia glutinosa* [21]. In a study investigating the growth of *Lactuca sativa* under different LED lights, it was observed that the fresh weight of the plant was higher when exposed to a combination of red and blue light compared to other treatments [47]. Furthermore, the yield of *Mentha piperita* was found to be higher when subjected to a combination of red and blue light than under the red light alone, suggesting a complementary effect of blue light on plant growth improvement [48]. However, it is important to note that the complementary effect of blue light may vary depending on the plant's genotype.

Light plays a crucial role in plant growth and development, and extensive research has been conducted to investigate the effects of different light qualities on plants. The growth rate of plants in conditions of low light or absolute darkness is faster and the plant becomes longer (etiolated), while in conditions of sufficient light, the growth of the plant is slower and becomes shorter. This phenomenon is influenced by the plant's ability to respond to ambient light, which is essential for completing the life cycle and optimizing performance. Light serves as a regulator and optimizer for various plant processes, including photosynthesis, biomass accumulation, stomata opening and closing, and structural changes in plant architecture structure [43,44,[49], [50], [51], [52]]. The effects of light on plants are complex and can vary depending on the specific species and their genetic characteristics. Numerous studies have highlighted the significant impact of different light spectra on plant growth, with red and blue light being particularly influential [53,54].

### 3.2 Light quality effects on chlorophylls and carotenoids contents

**Fig. 2:**
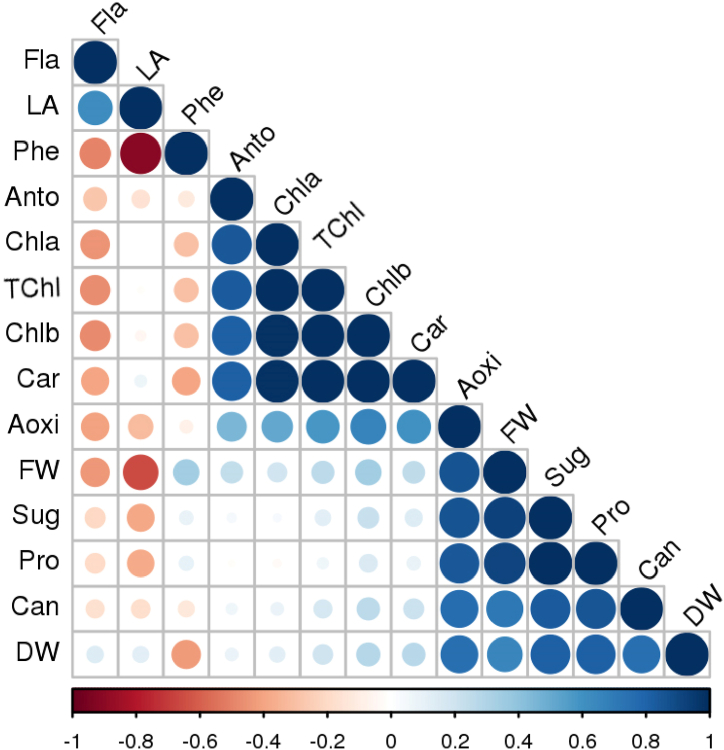
Correlation of physiological studied parameters in *T. vulgaris* (Abbreviations: Fla (Flavonoids); LA (Leaf Area); Phe (Total Phenol); Anto (Anthocyanin); Chla (Chlorophyll *a*); Chlb (chlorophyll *b*); TChl (Total chlorophyll); Car (Carotenoids); Aoxi (Antioxidant); FW (Plant Fresh Weight); Sug (Soluble Sugar); Pro (Proline); Can (Canopy); DW (Plant Dry Weight)).

Chl and carotenoid pigments effectively absorb red and blue light. Chl, an important pigment found in plant chloroplasts, plays a crucial role in light absorption for photosynthesis, which releases the absorbed energy to the next steps to the subsequent complex processes [[55], [56], [57]]. Fig. 2 illustrates the correlation analysis of various physiological parameters in *T. vulgaris* plants cultivated under different LED lighting conditions. The results demonstrate a positive correlation between antioxidant levels, soluble sugars, and proline, while revealing a negative correlation with flavonoids. Similarly, there is a positive correlation observed among antioxidant activity, chlorophyll *a*, chlorophyll *b*, total chlorophyll content, carotenoids, and anthocyanin, with a simultaneous negative correlation with flavonoids. These correlations emphasize the interconnected nature of physiological traits within *T. vulgaris* plants.

**Fig. 3:**
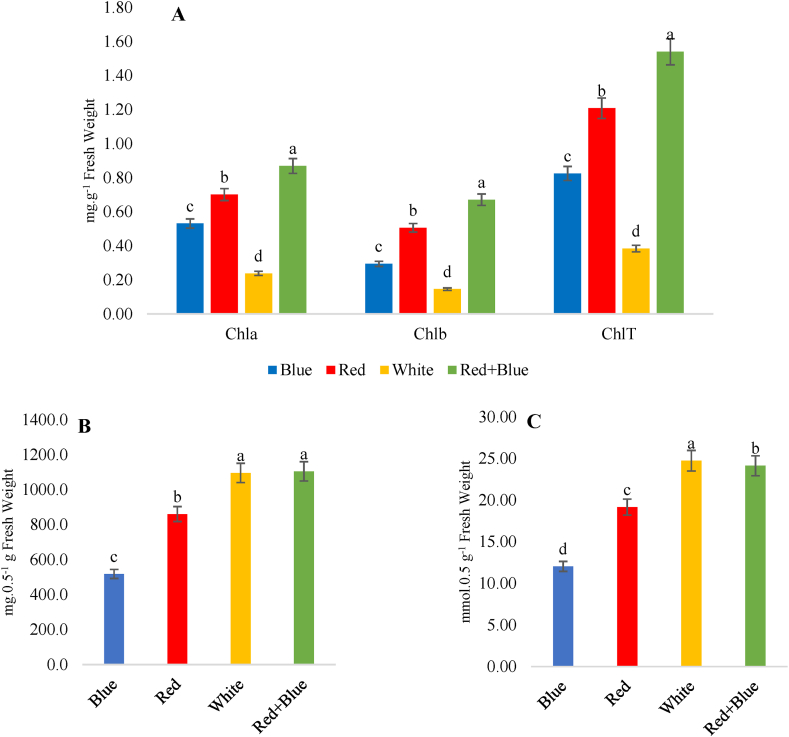
Influence of different light treatment on (A) chlorophylls, (B) soluble sugar and (C) proline of *T. vulgaris*. Different letters indicate statistical differences (P ≤ 0.01) according to an LSD test. Data are the average of three replications ± SE.

**Fig. 4:**
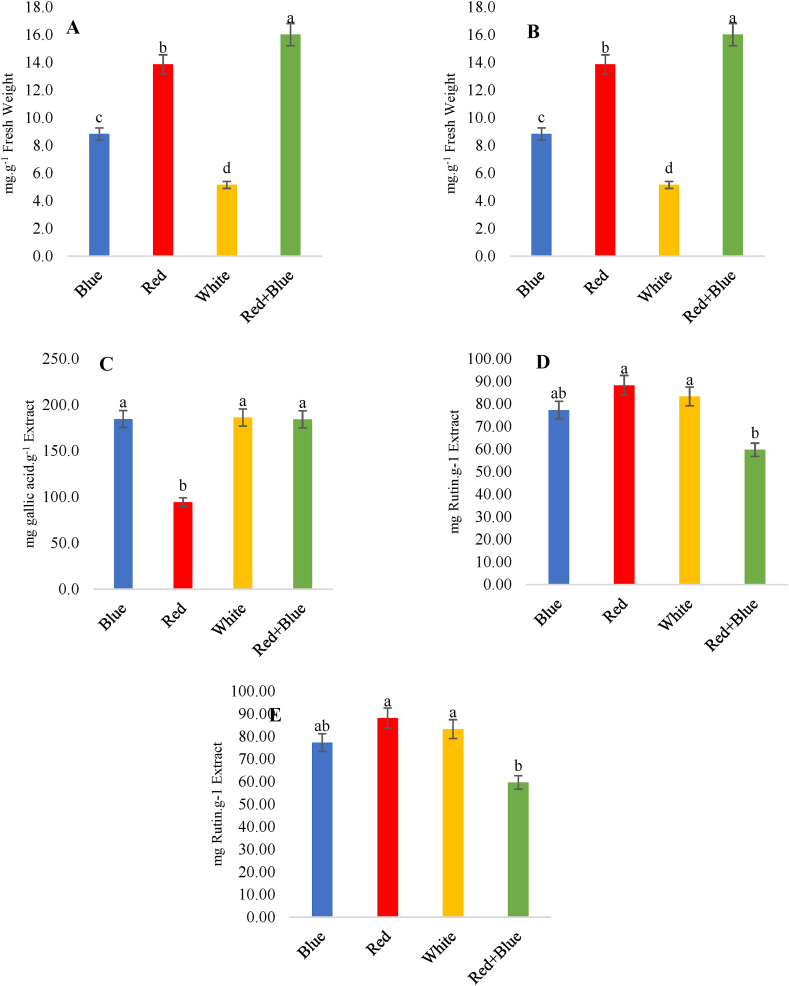
Effect of different light spectra on (A) carotenoids, (B) anthocyanin, (C) total phenolic compounds, (D) flavonoid and (E) antioxidant activity of *T. vulgaris*. Different letters indicate statistical differences (P ≤ 0.05) or (P ≤ 0.01) according to an LSD test. Data are the average of three replications ± SD.

The highest amounts of Chl a, b, and TChl as well as carotenoids of the studied samples were obtained under the red + blue light treatment with the values of 0.86, 0.76, 1.53, and 16.02 mg g^−1^ FW, respectively (Table 2; Fig. 3A; Fig. 4A). In contrast, the lowest amounts were observed under the white light treatment, with values of 0.23, 0.14, 0.38, and 5.14 mg g^−1^ FW, respectively. Previous reports have also indicated that the highest amounts of Chl a, Chl b, and carotenoids were observed under red light and red + blue light treatments [44,58,59].

**Table 2:** Influence of different light quality on physiologic and phytochemical traits of *Thymus vulgaris*.

	unit	Light quality			
		Blue	Red	White	Blue + Red
Chlorophyll *a* **	mg.g^−1^Fw	0.53 ± 0.04^c^	0.70 ± 0.04^b^	0.24 ± 0.03^d^	0.87 ± 0.01^a^
Chlorophyll *b* **	mg.g^−1^Fw	0.29 ± 0.03^c^	0.51 ± 0.01^b^	0.15 ± 0.03^d^	0.67 ± 0.02^a^
Total chlorophyll **	mg.g^−1^Fw	0.83 ± 0.01^c^	1.21 ± 0.01^b^	0.38 ± 0.01^d^	1.54 ± 0.01^a^
Carotenoids **	mg.g^−1^Fw	8.83 ± 0.05^c^	13.89 ± 0.05^b^	5.15 ± 0.05^d^	16.02 ± 0.17^a^
Soluble sugar **	mg.0.5 g^−1^Fw	518.60 ± 3.56^c^	860.50 ± 5.83^b^	1096.20±7^a^	1105.30 ± 14.01^a^
Proline **	Mmol.0.5 g^−1^Fw	12.03 ± 0.2^d^	19.16 ± 0.2^c^	24.74 ± 0.25^a^	24.13 ± 0.36 ^b^
Total phenol **	mg.GAE g^−1^ex	184.50 ± 5.83^a^	94.26 ± 3.71^b^	186.09 ± 5.78^a^	184.21 ± 9.61^a^
Flavonoid *	mg.Rutin g^−1^ex	77.27 ± 4.43^a^	88.20 ± 2.36^a^	83.27 ± 15.74^a^	59.67 ± 2.62^b^
Anthocyanin *	mg.g^−1^ex	5.09 ± 0.39^bc^	5.55 ± 0.17^ab^	3.12 ± 1.56^c^	7.31 ± 0.8^a^
Antioxidant activity **	μg.ml^−1^	16.35 ± 0.45^c^	19.46 ± 0.34^b^	19.20 ± 0.05^b^	21.87 ± 0.09^a^

Data are means ± SE (n = 3).

Ex: extract; GAE: Gallic acid.

ns: Values are not significantly different.

*: Values are significantly different at *P* ≥ 0.05.

**: Values are significantly different at *P* ≥ 0.01.

The values followed by the same alphabet do not significantly differ according to Fisher's Duncan test.

The light spectrum has a significant impact on the internal structure of chloroplasts [60]. In *Cannabis sativa*, the highest Chl content was obtained under blue light conditions [38]. Similar results have been reported in lettuce, highlighting the positive effect of blue light on Chl accumulation [61]. Blue light has been found to significantly increase the Chl content and gas exchange rate in plant leaves. However, conflicting reports exist regarding the increasing or decreasing effects of blue light, suggesting variations in plant responses [38]. Conversely, the rate of photosynthesis was found to decrease in rice leaves grown under red light [62]. The quality of light directly influences photosynthesis by affecting the content and types of chlorophyll pigments. In the published report on the effect of different light spectrums on *Isochrysis zhangjiangensis*, the highest amount of Chl was observed in green light and the lowest amount in blue light [63]. These results indicate that the type of light spectrum affects the accumulation of photosynthetic pigments. Additionally, a negative correlation between the Chl a/b ratio and the percentage of red to blue light usage has been observed. In *Cattleya loddigesii* Lindl, it was shown that red light strongly enhances Chl b synthesis [64]. Similar results have been reported in *Rehmannia glutinosa* Libosch and *Triticum aestivum* plants [21,61]. In *Lactuca sativa*, the Chl content was found to be higher under blue light treatment than under red light conditions [61]. In some medicinal plants, Chl biosynthesis predominantly takes place in blue light more than in red light. The presence of red light can decrease the production of the precursor 5-aminolevulinic acid, leading to a decline in Chl synthesis [65,66].

The present study revealed significant differences in carotenoid content when applying different light spectra. The highest carotenoid content was obtained in the treatment of red + blue light (16 mg/g FW) and red light (13.9 mg/g FW), while the lowest content of this pigment was observed in white light (5.1 mg/g FW). In stress conditions, carotenoids as antioxidants play an important role in various plant processes. They act as a photosynthetic auxiliary pigment in absorbing excess light or ultraviolet rays, scavenging reactive oxygen species and dispersing excess energy. Under abiotic stress, the production of carotenoids increases [67,68]. In a study conducted on Basil, Fenugreek, Dill, and Chervil, significant differences in carotenoid content were observed among the studied species as well as different optical spectra. The highest accumulation was measured in Fenugreek (0.058 mg g1 FW), while the lowest was found in Dill (0.015 mg g1 FW) [65]. Similar studies conducted on Triticum aestivum [69,70], *Raphanus sativus*, *Lactuca sativa*, and *Spinacia oleracea* [45,71,72] have reported identical results, indicating an increase in carotenoid content under red + blue light.

### 3.3 Light quality effects on proline and soluble sugar

In terms of proline accumulation, the highest amounts were observed under the white and red + blue light treatments, with values of 24.73 and 24.13 mmol.0.5 g^−1^ of the leaf tissue, respectively, while the lowest amount obtained 12.02 mmol.0.5 g^−1^ under the blue light treatment (Table 2, Fig. 3C). In contrast to our findings, some studies have reported maximum proline contents in plants grown under blue and orange light.

In contrast to our findings, some studies have reported maximum contents of proline in plants grown under blue and orange light, respectively, such as in *Brassica napus* L., where proline levels reached 36 and 40 μg g^−1^ (FM), respectively, compared to white light [73]. Similarly, studies conducted on the Chrysanthemum plant and *Prunella vulgaris* showed that the highest proline content was obtained in blue light treatment [31,74]. The impact of blue light exposure on proline and soluble sugar concentrations can vary due to experimental conditions, plant species, growth stage, genetic variability, and duration of exposure. Even slight variations in these factors can lead to divergent outcomes. For instance, García-Caparrós et al. [75], revealed that the response of *Chlorophytum comosum* and *Tradescantia zebrina* varied when it came to the concentration of total soluble sugars and proline when exposed to either monochromatic or mixed red-blue LEDs.

The highest content of soluble sugar was observed under the red + blue and white light treatments (Fig. 3B), with the values of 1105.3, and 1096.2 mg ml^−1^ of the extract, respectively. The lowest content of soluble sugar was obtained under the blue light treatment, with a value of 518.6 mg ml^−1^ of the extract. Sugars play a crucial role in regulating osmosis as well as maintaining the stability of membranes and proteins. A study on *Brassica alboglabra* Bailey found that plants exposed to the 8R1B light treatment exhibited significantly higher levels of sucrose, glucose, and fructose compared to the white light treatment, which correlated with improved plant growth and biomass, highlighting the positive link between increased sugar content and enhanced plant development [76].

In this regard, Chen et al. [60] notified an increase in the soluble sugar content of lettuce under the combination of red and blue light compared to red or blue monochromatic light. Based on our findings, a combination of red and blue light or white light is recommended as a stress factor because the production of proline, soluble sugars, and polyphenols in plants could be part of a stress alternative strategy [77,78].

Blue light exposure in the present study resulted in the lowest concentrations of proline and soluble sugars. This suggests that blue light may not cause serious stress for plants in terms of these metabolic parameters. Previous studies have shown that red light is beneficial for the accumulation of soluble sugars, while blue light promotes the accumulation of soluble proteins [2,79,80]. This indicates that different light wavelengths have distinct effects on sugar and protein metabolism in plants. The combination of red and blue light has been found to increase both soluble sugars and proteins, suggesting that an appropriate mixture of these wavelengths can enhance the accumulation of these important building blocks for plant growth [81]. When considering the average plant weight, it is evident that the red and blue light treatment yielded the highest weight, while the blue light treatment resulted in the lowest weight. This indicates that the red and blue light combination may have a positive influence on plant growth, while blue light alone may be less optimal for promoting plant weight. Several studies have demonstrated that red and blue light combinations are generally more effective in promoting plant growth and weight compared to monochromatic light sources or other light treatments [1,[82], [83], [84], [85]].

### 3.4 Light quality effects on phenols, flavonoids, and anthocyanin

The highest amount of phenolic compounds was 186.08 mg gallic acid per gram of the dry extract under the white light treatment, while the lowest amount was obtained under the red light treatment with a value of 94.26 mg/g extract (Fig. 4C). Similarly, the highest and lowest amounts of flavonoids were 88.2 and 59.66 mg g^−1^ extract, respectively obtained under the red and red + blue light condition (Table 2; Fig. 4D). Phenolic compounds generally serve as defensive compounds against herbivores, pathogens, mechanical damage, harmful ultraviolet radiation, and for attracting pollinators [86]. The biosynthesis of phenolic compounds relies on light, and their production rate is influenced by light intensity [87]. Kim et al. [88] have emphasized that light stimulates the accumulation of phenolic compounds by increasing the production of Malonyl CoA and Coumaroyl CoA, which are precursors for phenolic compound biosynthesis [88,89]. Thus, the application of complementary red or blue light may increase the accumulation of phenolic compounds in some plant species; however, its increasing effects depend on the species and specific compounds [90]. Polyphenols, as plant phytochemical compounds, play a vital role in protecting plants against adverse environmental changes, as plants are unable to move or escape from various stresses [[91], [92], [93]].

Chicoric acid, the second major phenolic compound in *Ocimum basilicum*, exhibited the highest concentration under white light treatment, followed by the combined blue and red light conditions. The studied plants were exposed to 100 μmolm^−2^s^−1^ PPFD for 14 days [94,95]. Several studies have reported an increase in phenols and antioxidant activity under complementary blue light conditions in medicinal species such as *Kalanchoe pinnata* [96].

Flavonoids are known for their important role in protecting against light damage [97]. The increase in flavonoid concentrations may be attributed to the high activity of phenylalanine ammonia-lyase (PAL) or the rapid synthesis of this enzyme under various factors, including UV stress [98,99]. Manivannan et al. [21] reported that the total flavonoid amount in *Rehmannia glutinosa* is increased under the red light condition. Red light has been found to stimulate quercetin production (a flavonoid) in Pisum sativum through the activation of stress-modulating production or phytochromes that modulate flavonoids [100]. Similar events likely occurred in *T. vulgaris* under red light treatment, leading to the accumulation of flavonoid compounds.

Regarding the impact of light quality on anthocyanin compounds (Fig. 4B), the highest accumulation rate of 7.3 mg g^−1^ extract was observed under red + blue light conditions, whereas the lowest accumulation rate of 3.12 mg g^−1^ extract was recorded under white light treatment. Nicole et al. [6] have highlighted that the combination of blue and red light increases the extent of lettuce leaf anthocyanin compared to the application of only red light.

In recent decades, the role and function of anthocyanins, one of the most important pigments in plant leaves, have been extensively studied [101,102]. Environmental stresses such as low temperatures, inappropriate lighting, and nutrient deficiencies can enhance anthocyanin levels in leaves. Under high light conditions, chemical compounds such as anthocyanins and carotenoids accumulate to reduce the energy from wavelengths. However, the effects of these stresses vary among plant species. Anthocyanins provide protection against light damage, particularly UV light [103]. Anthocyanins upregulated by cryptochromes to regulate anthocyanin production [104]. the regulation of anthocyanin production in plants involves the cooperation of multiple photoreceptors, including cryptochromes and phytochromes. While cryptochromes are involved in this process, their full regulatory capacity in anthocyanin production relies on the presence of active phytochrome. Active phytochrome signaling, triggered by red light, enhances the sensitivity of cryptochromes to blue light, amplifying their regulatory effects on anthocyanin-related genes [105,106]. This interplay between cryptochromes and phytochromes ensures a coordinated response to different light qualities and intensities, ultimately influencing the biosynthesis of anthocyanins in plants [107,108].

### 3.5 Light quality effects on antioxidant activity

The highest amount of IC50 was obtained in the red + blue light with a rate of 21.86 μg ml^−1^ (Fig. 4E). Nonetheless, along with an increase in this rate, the antioxidant activity decreased and the lowest amount of IC50 was observed in the blue light treatment with a value of 16.34 μg ml^−1^, indicating high antioxidant activity (Table 2). Since the biosynthesis of phytochemical compounds is affected by light quality, antioxidant activity is likely to be increased due to a change in the light spectrum. Previous research has shown that red, blue, and UV lights, compared to white or sunlight, increase the concentration of EO content, antioxidant activity, and phenolic compounds [90].

Consistent with our findings, Dou et al. indicated that blue light significantly increases antioxidant activity [90]. Kim et al., also showed that the amount of antioxidant content in cherry tomatoes doubled under blue light [3]. Ouzounis et al., in their experiment on *Rosa hybrida*, *Chrysanthemum morifolium*, and *Campanulus portenschlagiana*, observed that compared to white light, the use of monochromatic red and blue lights for four weeks significantly increased the antioxidant capacity and flavonoid content [109]. In conformity with this research, Manivannan et al. [21] showed that monochromatic red and blue LED lights, compared to the white light from a fluorescent lamp, increased both antioxidant and flavonoid capacities in *Rehmannia glutinosa*. The presence of a high amount of reactive free radicals in the human body can lead to oxidative damage and various diseases [110]. The key role of anthocyanins and antioxidants in protecting against oxidative stress has been proven by previous researchers [111].

Moreover, phenols possess structural properties that enable them to scavenge free radicals and prevent oxidative damage. Therefore, higher phenolic content enhances the antioxidant capacity in plants [112]. Plants with high phenolic content and antioxidant capacity are desired for commercial and medicinal purposes [113]. Complementary blue light has been associated with increased phenol content and antioxidant activity [96]. Although complementary blue light may not necessarily increase the total phenol content in strawberry fruits, it can enhance antioxidant activity and accelerate fruit ripening [114].

Based on our study and the references mentioned, it can be inferred that proline has positive effects under light stress conditions, particularly in enhancing antioxidant activity and mitigating the negative effects of UV-B radiation [3,90,115]. The studies mentioned earlier indicate that blue light exposure significantly increases antioxidant activity and the accumulation of antioxidants, such as flavonoids, in plants. Proline, as a precursor for antioxidant molecules, plays a role in enhancing the antioxidant capacity of plants. The content of proline tends to increase in plants under various environmental stresses [116].

In the study on lettuce, pre-treatment with proline was found to alleviate the adverse effects of UV-B radiation through multiple mechanisms, including increased activity of antioxidant enzymes, higher levels of phenolic compounds, upregulation of defense-related genes, modulation of growth regulators, and accumulation of soluble sugars and organic acids. These effects collectively contributed to the plant's ability to counteract UV-B stress. Therefore, Proline demonstrates potential as a protective agent against light stress, including UV-B radiation, by enhancing antioxidant activity and promoting various protective mechanisms in plants [117].

### 3.6 Light quality effects on EO compounds

Table 3 presents the ten identified monoterpene compounds in the *T. vulgaris* EO profile. Notably, thymol and carvacrol compounds were not observed in this study. Instead, high values were found for *p*-Cymene and *γ*-Terpinene, which are precursors of thymol and carvacrol biosynthesis. When either *γ*-Terpinene or *p*-Cymene hydrocarbons are the main constituents of the EO, they are referred to as phenolic EOs. Hence, there is a direct relationship between the levels of these compounds and the extent of phenol and antioxidant activity. The data revealed that *p*-Cymene exhibited the highest values in the white and blue light treatments, with values of 60.92 % and 59.53 %, respectively. Likewise, under the same light conditions, phenol content and antioxidant activity were significantly high. Moreover, *γ*-Terpinene content was highest in the red + blue light treatment, with a value of 15.43 %. Most of the identified volatile compounds belonged to the group of monoterpenes. It has been revealed that environmental stimuli such as temperature, seasonal variation, photoperiod, and light intensity effectively influence the biosynthesis and accumulation of monoterpenoid components, as specialized metabolites [118,119].

**Table 3:** The influence of different light qualities on the volatile compound of *Thymus vulgaris*.

Compound	Phytochemical group^a^	KI^b^	KI^c^	Percentage of compounds			
				Blue + Red	White	Red	Blue
2-Methylbutanoic acid, methyl ester^e^	C	778	768	4.37 ± 0.56^a^	1.2 ± 0.52^b^	1.71 ± 0.51^b^	3.8 ± 0.2^a^
α-Thujene^e^	CM	932	932	6.71 ± 0.71^a^	5.59 ± 0.61^ab^	5.3 ± 0.5^b^	4 ± 0.6^c^
α-Pinene^d^	CM	941	941	6.21 ± 0.84^a^	6.52 ± 0.41^a^	6.84 ± 0.76^a^	4.53 ± 0.71^b^
Camphene ^ns^	CM	960	960	4.28 ± 0.71^b^	5.31 ± 0.62^ab^	5.96 ± 1.04^a^	4.14 ± 0.81^b^
β-Pinene^e^	CM	990	990	–	0.52 ± 0.17^a^	–	–
Myrcene^e^	LM	997	995	2.35 ± 0.08^a^	0.99 ± 0.13^c^	1.53 ± 0.26^b^	1.78 ± 0.32^b^
α-Terpinene^e^	CM	1032	1030	1.87 ± 0.21^a^	1.22 ± 0.46^b^	2.24 ± 0.35^a^	0.99 ± 0.25^b^
*p*-Cymene^d^	CM	1046	1045	48.89 ± 5.50^c^	60.92 ± 4.41^a^	51.53 ± 3.9^bc^	59.53 ± 3.44^ab^
γ-Terpinene^e^	CM	1076	1075	15.43 ± 1.36^a^	7.47 ± 0.85^c^	13.09 ± 1.38^b^	11.2 ± 1.05^b^
Linalool^d^	LM	1130	1112	1.96 ± 0.41^ab^	2.37 ± 0.5^a^	1.1 ± 031^b^	2.74 ± 0.65^a^
Thymol acetate ^ns^	CM	1378	1362	1.08 ± 0.33^ab^	0.76 ± 0.23^b^	1.17 ± 0.25^ab^	1.46 ± 0.34^a^
β-Caryophyllene ^ns^	S	1454	1455	1.14 ± 0.26^ab^	1.09 ± 0.27^ab^	1.72 ± 0.42^a^	1.05 ± 0.32^b^
Total				94.29	93.96	92.19	95.22

ns: Values are not significantly different.

The values followed by the same alphabet do not significantly differ according to Fisher's Duncan test.

a Carboxylic acid (C), Cyclic Monoterpenes (CM), Linear Monoterpene (LM), Sesquiterpen (S).

b calculated KI index.

c reference KI index; Data are means ± SE (n = 3).

d Values are significantly different at *P* ≥ 0.05.

e Values are significantly different at *P* ≥ 0.01.

Insert Table 3 linalool is produced in plants by the linalool synthase enzyme from the general precursor of monoterpenes, geranyl diphosphate, and through the myrcene/osmium synthase enzyme from myrcene. The highest and lowest extent of linalool were observed in the blue (2.74 %) and the red (1.10 %) light treatments, respectively. *α*-Phellandrene and *β*-Pinene compounds in plants are produced by *α*-terpineol synthase, 1-8-cineole synthase, and *α*/*β* pinene synthase enzymes from geranyl diphosphate, respectively. *β*-pinene was not found in the red or blue monochromatic or red + blue combination lights; nevertheless, it was present in the white light condition, indicating that all light spectra are required for its expression.

Zhang et al. reported that sunlight exclusion greatly inhibits the biosynthesis of linalool and ocimene as the free volatiles in grape exocarp and mesocarp [120]. The mechanism responsible for inducing and accumulating certain specialized compounds of the thyme plant under monochromatic red and blue spectra, as well as combined red + blue or white spectra, is not yet clear. However, it is likely that these wavelengths are related to the activation of specific plant genes that ultimately promote the increase in specialized metabolites [121]. Moreover, it is highly likely that the enzymes' function in the pathway of specialized compounds formation is affected by various light spectra. Compared to other lights, the blue light regime resulted in the highest production of specialized metabolites per gram of the dried samples.

In this study, it was confirmed that the primary and specialized metabolites of *T. vulgaris* vary in response to different wavelengths generated by LEDs. Thus, studying the effective factors on plant growth and examining their strong impact on the development of specialized metabolites is very important for the pharmacology and medicalization of the Thyme plant. While light is one of the most influential regulators of specialized metabolites, further research is needed to understand how plants adapt to the compounds accumulated under different light spectra [109].

### 3.7 Pearson correlation and principal component analysis

**Fig. 5:**
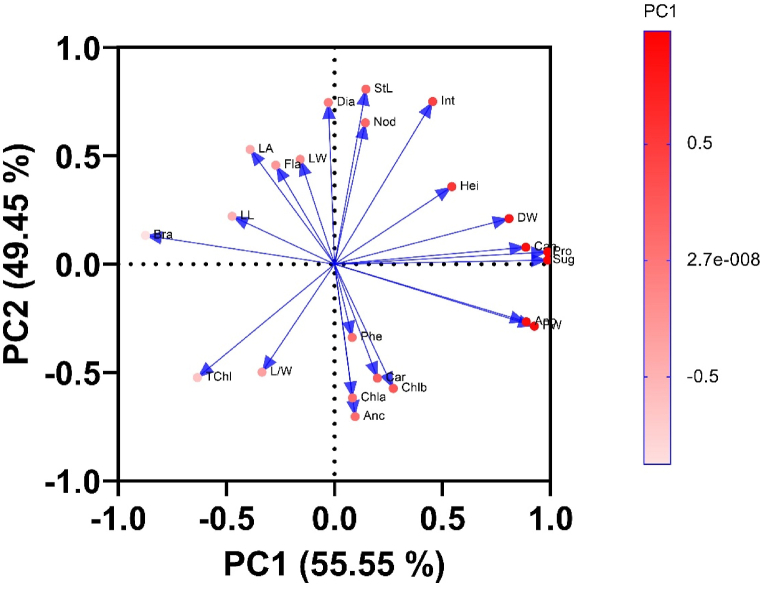
Principal Component Analysis (PCA) of *T.vulgaris* traits in different light qualities. (Abbreviations: Fla (Flavonoids); LA (Leaf Area); Phe (Total Phenol); Anc (Anthocyanin); Chla (Chlorophyll *a*); Chlb (chlorophyll *b*); TChl (Total chlorophyll); Car (Carotenoids); Ano (Antioxidant Activity); FW (Plant Fresh Weight); Sug (Soluble Sugar); Pro (Proline); Can (Canopy); DW (Plant Dry Weight); Bra (Number of Branch); L/W (Leaf Length/Leaf Width); LL (Leaf Length); Leaf Width (LW); Int (Internode length); Nod (Number of Node); StL (Stem Length); Dia (Stem Diameter); Hei (Plant Height)).

The correlation between growth parameters (traits that had significant differences between light quality treatments), Chl, and antioxidant content is shown in Fig. 2. As can be seen, a significant positive correlation has been shown between anthocyanin content with Chla, Chlb, and carotenoids. Chl content also showed a significant positive correlation with carotenoid content. Additionally, a significant positive relationship was observed between antioxidant activity and anthocyanin, carotenoids, fresh weight, dry weight, canopy width, proline, and sugar content. Leaf surface area displayed a significant negative relationship with phenolic compound content and fresh weight. To further investigate the effects of different light spectra on the evaluated traits of *T. vulgaris*, Principal Component Analysis (PCA) loading diagrams were generated and are presented in Fig. 5.

### 3.8 Strength and limitations

Our study significantly contributes to the existing knowledge regarding the mechanism of how light affects plant development. Prior to our research, the specific effects of different light spectra on the growth and biochemical processes of *T. vulgaris* were largely unknown. Our study fills this knowledge gap by providing valuable insights into the morphological characteristics, physiological traits, and primary and specialized metabolites. For instance, our results demonstrate that specific light conditions, such as blue and white light treatment, significantly enhance the levels of some important compounds such as phenols and antioxidants. This information is particularly relevant for industries relying on *T. vulgaris* for pharmaceutical and medical purposes.

However, there are some limitations in the present study which should be noted. Firstly, our study was conducted with a limited sample size, which may affect the generalizability of the results although we believe that the significant differences observed in the measured parameters provide valuable insights into the effects of different light spectra on *T. vulgaris*. Also, we focused on the effects of different light spectra, but we did not investigate the influence of light intensity and duration. These factors could potentially interact with the light spectra and affect the observed outcomes. Future studies should consider exploring the combined effects of light intensity, duration, and spectra.

## 4 Conclusion

The findings of this study revealed that changing the light conditions leads to changes in growth parameters and specialized metabolites. Also, the antioxidant activity and phenolic contents, which are very important for the pharmaceutical and medical industry, were underlined to be the highest under the blue light treatment. Moreover, the measurement of Chl, carotenoids, soluble sugar, proline, and anthocyanins indicated that the red + blue light may be identified as a stress-generating factor in *T. vulgaris*. Finally, according to the results of this study, it seems that different wavelengths can be used to purposefully increase primary and specialized metabolites or even achieve the desired growth characteristics.

## Data availability Statement

The Data included in article/supp. Material/referenced in article can allow other scholars to reuse these data on the following Links:

Research Gate:

https://www.researchgate.net/profile/Forouh-Sadat-Seyedi.

LinkedIn:

https://www.linkedin.com/in/forouh-sadat-seyedi-0890692a0/

## CRediT authorship contribution statement

**Forouh Sadat Seyedi:** Writing – review & editing, Writing – original draft, Investigation, Formal analysis. **Mehdi Ghasemi Nafchi:** Writing – review & editing, Writing – original draft, Funding acquisition, Conceptualization. **Saeed Reezi:** Writing – review & editing, Writing – original draft, Validation, Methodology, Funding acquisition, Conceptualization.

## Declaration of competing interest

The authors declare the following financial interests/personal relationships which may be considered as potential competing interests.
